# Microbial ‘Old Friends’, immunoregulation and stress resilience

**DOI:** 10.1093/emph/eot004

**Published:** 2013-04-09

**Authors:** Graham A. W. Rook, Christopher A. Lowry, Charles L. Raison

**Affiliations:** ^1^Centre for Clinical Microbiology, Department of Infection, University College London (UCL), London, UK; ^2^Department of Integrative Physiology and Center for Neuroscience, University of Colorado Boulder, Boulder, CO 80309-0354, USA and ^3^Department of Psychiatry, College of Medicine and Norton School of Family and Consumer Sciences, College of Agriculture and Life Sciences, University of Arizona, Tucson, AZ, USA

**Keywords:** immunoregulation, depression, microbial ‘Old Friends’, stress resilience, chronic inflammatory disorders

## Abstract

Chronic inflammatory diseases (autoimmunity, allergy and inflammatory bowel diseases) are increasing in prevalence in urban communities in high-income countries. One important factor is reduced exposure to immunoregulation-inducing macro- and microorganisms and microbiota that accompanied mammalian evolution (the hygiene hypothesis or ‘Old Friends’ mechanism). Reduced exposure to these organisms predisposes to poor regulation of inflammation. But inflammation is equally relevant to psychiatric disorders. Inflammatory mediators modulate brain development, cognition and mood, and accompany low socioeconomic status and some cases of depression in developed countries. The risk of all these conditions (chronic inflammatory and psychiatric) is increased in urban versus rural communities, and increased in immigrants, particularly if they move from a low- to a high-income country during infancy, and often the prevalence increases further in second generation immigrants, suggesting that critical exposures modulating disease risk occur during pregnancy and infancy. Diminished exposure to immunoregulation-inducing Old Friends in the perinatal period may enhance the consequences of psychosocial stressors, which induce increased levels of inflammatory mediators, modulate the microbiota and increase the risk for developing all known psychiatric conditions. In later life, the detrimental effects of psychosocial stressors may be exaggerated when the stress occurs against a background of reduced immunoregulation, so that more inflammation (and therefore more psychiatric symptoms) result from any given level of psychosocial stress. This interaction between immunoregulatory deficits and psychosocial stressors may lead to reduced stress resilience in modern urban communities. This concept suggests novel interpretations of recent epidemiology, and novel approaches to the increasing burden of psychiatric disease.

## INTRODUCTION

Inflammation is a protective mechanism, classically accompanied by pain, heat, redness and swelling, that evolved to remove tissue-damaging stimuli (such as infections) and then initiate the healing process. Negative feedback mechanisms exist to block inappropriate inflammatory responses such as those targeting self or gut contents, and to terminate inflammation when it is no longer required. However, these regulatory mechanisms increasingly fail. The high-income countries have undergone massive increases in the prevalence of a wide range of chronic inflammatory disorders including allergies, autoimmune diseases and inflammatory bowel disease (IBD), where the inflammatory responses are both inappropriate and not terminated. Rigorous meta-analyses have confirmed that these increases are real, and not artifacts due to changing diagnostic criteria [1, 2]. The increases correlate with economic development and urbanization, and the start of the process in Europe can be traced back to the 19th century when it was noted that hay fever was rare in farmers, and characteristic of rich urban educated people [3, 4]. Recent studies have confirmed the protective effect of the farming environment [5–7] and shown that contact with animals such as dogs is also protective [8]. In addition to these observations on allergic disorders, a link between lifestyle and an autoimmune disease was explicitly suggested in 1966, when it was reported that the prevalence of multiple sclerosis (MS) showed a positive correlation with sanitation in Israel [9]. However, it was not until 1989 that the term ‘Hygiene Hypothesis’ was coined following the observation that in young adults a history of hay fever was inversely related to the number of siblings (especially older male siblings) in their family when they were 11 years old [10]. Then, Matricardi *et al.* [11] found that army recruits with evidence of infections attributable to fecal–oral transmission were less likely to have allergic manifestations. Such data were considered consistent with a protective influence of postnatal infection that might be lost in the presence of modern hygiene [10–12]. A few years later it was pointed out that Type 1 diabetes (T1D; caused by autoimmune destruction of the insulin-secreting β cells in the pancreas) is increasing at the same rate, and in the same countries (mostly high income) as the allergic disorders [13]. Similarly, a parallel rise in IBDs (Crohn’s disease (CD) and ulcerative colitis (UC)) had clearly started at the beginning of the 20th century, rising from rare and sporadic in 1900, to 400–500/100 000 by the 1990s in high-income countries [2].

In this review, we discuss the increasing evidence that much of the failure of regulation of inappropriate inflammatory immune responses in people living in modern cities in high-income countries is attributable to progressive loss of contact with organisms with which we co-evolved and that play a crucial role in setting up the regulatory pathways (the Old Friends mechanism). We then discuss the evidence that some psychiatric disorders might be increasing for the same reasons. We point out that the epidemiology of chronic inflammatory disorders and psychiatric disorders show parallels in relation to urban–rural differences and the effects of immigrant status. We then show how the Old Friends mechanism operating in the perinatal period can synergize with psychosocial stressors to drive long-term defects in immunoregulation. Finally, we suggest that these mechanisms lead to novel interpretations of some published work, including the health deficits associated with gradients of socioeconomic status (SES).

## THE OLD FRIENDS MECHANISM AND IMMUNOREGULATION

**Figure 1.**
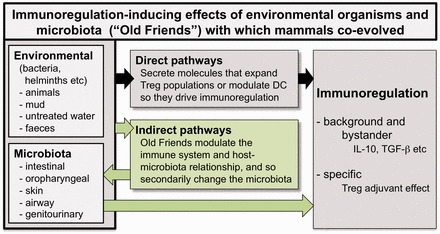
Microbial immunomodulation. Microbes from the environment, and from the various microbiota, modulate the immune system. Some of this is due to direct effects of defined microbial products on elements of the immune system. But modulation of the immune system also secondarily alters the host–microbiota relationship and leads to changes in the composition of the microbiota, and so to further changes in immunoregulation (shown as indirect pathways)

The recent increases in chronic inflammatory disorders are at least partly explained by the Hygiene Hypothesis or by the variant of that hypothesis that we prefer, the ‘Old Friends’ mechanism, operating in synergy with other factors discussed later. The Old Friends mechanism states that mammals co-evolved with an array of organisms that, because they needed to be tolerated, took on a role as inducers of immunoregulatory circuits [14, 15]. Such organisms include various microbiotas and commensals (gut, skin, lung, etc.); chronic infections picked up at birth; helminths that persist for life and environmental organisms from animals, mud and untreated water with which we were in daily contact in the environments in which humans evolved and lived until recently (Fig. 1). For example, helminthic parasites need to be tolerated because although not always harmless, once they are established in the host, the immune system is incapable of eliminating them. In patients with blood nematode infections, the inflammatory response is downregulated to avoid excessive tissue damage [16]. When such downregulation fails elephantiasis results [16]. Contact with the immunoregulatory ‘Old Friends’ rapidly diminishes when industrialization occurs, and individuals start to inhabit a plastic and concrete environment, to consume washed food and chlorine-treated water, and to minimize their contact with mud, animals and faeces. This withdrawal of the organisms that drive immunoregulatory circuits results in defective immunoregulation that, depending on the genetic background of any given individual, can manifest as a variety of chronic inflammatory disorders, including allergies, IBD and autoimmunity. Early articulations of the hygiene hypothesis focused exclusively on allergic conditions, but we now know that a failure of immunoregulatory mechanisms really can lead to simultaneous increases in diverse types of pathology. For example genetic defects of the gene encoding the transcription factor Foxp3 lead to the X-linked autoimmunity–allergic dysregulation syndrome that includes aspects of allergy, autoimmunity and enteropathy [17]. Box 1 contains further background to the Old Friends mechanism, and some discussion of molecular mechanisms and relevant clinical trials.

**Box 1. The crucial evidence for the ‘Old Friends’ mechanism**

The crucial points of evidence supporting an association between chronic/inflammatory conditions and the absence of ‘Old Friend’ organisms from modern environments are these. First, the chronic inflammatory disorders all show evidence of failed immunoregulation [18]. Second, ‘Old Friends’ (such as helminths, non-pathogenic environmental bacteria [pseudocommensals] or certain gut commensals and probiotics) have been shown to drive immunoregulation, and to block or treat models of ‘all’ of these chronic inflammatory conditions [19–21]. Third, some Old Friends, or molecules that they secrete, can be shown to specifically expand populations of regulatory T cells (Treg) [21–24], or to cause dendritic cells to switch to regulatory phenotypes that preferentially drive immunoregulation [25]. Finally, when MS patients become infected with helminths, the disease stops progressing and circulating myelin-recognizing regulatory T cells (Treg) appear in the peripheral blood [26, 27], indicating that the helminths act as Treg adjuvants. This observation has led to formal clinical trials [28].

Many ‘Old Friends’ are (or were, until changed or depleted) gut microbiota, or gut parasites [22–24]. Others were environmental saprophytes in mud and untreated water that inevitably passed though the gut in large numbers every day [29]. Moreover, new data show that other microbiota such as those of the skin or oral mucosa can also be relevant to immunoregulation [30–32]. Thus, changes in the microbiota, which is profoundly different in Europeans than in people living in a traditional rural African village [33], must be regarded as part of the Old Friends hypothesis, whether these changes are attributable to diet [34] or to diminished exposures to the organisms themselves. In either case, altered exposure to ‘Old Friends’ will simultaneously exert direct effects on the immune system and indirect effects via secondarily induced changes in the microbiota.

### The Old Friends mechanism and high-income countries

The Old Friends mechanism implies that inflammation is better regulated in low-income than in high–income urbanized countries. At first sight this seems paradoxical, because the high prevalence of infections in low-income countries might be expected to cause high levels of inflammation [35]. However, recent work by McDade *et al.* [36] discussed previously in this journal [37] has largely resolved this paradox. The results reveal that in a low-income country where there is still abundant exposure to the immunoregulation-inducing ‘Old Friends’, immunoregulation is efficient, and the inflammatory response is vigorous during an infection, but it is terminated when no longer needed, with the result that ‘resting’ C-reactive protein (CRP) is close to zero. This longitudinal study illuminated the previous finding that ‘high’ levels of microbial exposure in the perinatal period and in infancy correlated with ‘low’ levels of ‘resting’ CRP in adulthood [38]. In contrast, in the USA and other high-income countries, there is often constant low-grade inflammation which tends to be stable across individuals, manifested as chronically raised CRP or interleukin (IL)-6, in the absence of any clinically apparent inflammatory stimulus. Such chronically elevated inflammation greatly increases the risk of subsequent inflammatory disease and cardiovascular problems and has been shown in some studies to predict the future development of depression [39].

## INFLAMMATION AND PSYCHIATRIC DISORDERS

**Figure 2.**
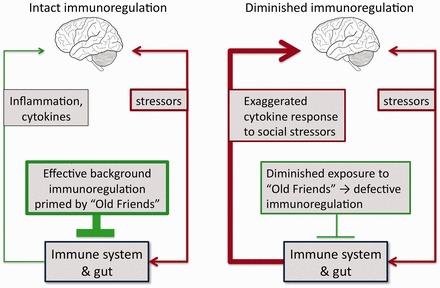
Immunoregulation and the inflammatory response to psychosocial stressors. Stress drives release of proinflammatory mediators via pathways that involve the immune system and the gut. The inflammatory response to a given level of a stressor is modulated and eventually terminated by immunoregulatory mechanisms. If immunoregulation is defective, as can occur when there has been inadequate exposure to immunoregulation-inducing Old Friends, then a given level of stressor will result in greater and more prolonged inflammatory response

Inflammation is involved not only in chronic inflammatory disorders such as allergies, autoimmunity and IBD but also in many psychiatric disorders. We have reviewed this topic in detail elsewhere [40, 41]. Briefly, a large subset of depressed individuals has persistently raised levels of proinflammatory cytokines and other downstream inflammatory markers [42, 43], together with a relative deficit in anti-inflammatory mediators and regulatory T cells [fully referenced in 41]. Interestingly, depressed individuals also show exaggerated release of inflammatory mediators in response to psychosocial stressors [44], implying altered immunoregulation (Fig. 2), and epidemiological studies in the UK showed that raised CRP and IL-6 predict subsequent risk of depression [39]. Similarly, people who respond to a laboratory stressor (the Trier Social Stress Test (TSST)) with negative emotionality and raised IL-1β are at increased risk of developing depression over the subsequent year. Detailed analysis indicated that the IL-1β was a significant mediator of this effect [45].

The possibility that inflammatory mediators might play direct causal roles in the development of psychopathology has been confirmed for interferon-alpha (IFN-α), IL-6 and tumor necrosis factor (TNF). When IFN-α is used therapeutically (to treat viral hepatitis or some cancers), it causes a high-rate of depression-like symptoms that respond to standard antidepressants, such as selective serotonin reuptake inhibitors [43, 46]. The dose of IFN-α is large, but in most studies, it is not a constant infusion but a weekly infusion with blood levels that peak then fall. Interestingly, IFN-α produces physiological abnormalities observed in major depressive disorder (MDD): alterations in hypothalamic–pituitary–adrenal (HPA) axis, sleep, monoamines, increased TNF and the development of these abnormalities correlates with the development of depressive symptoms [43, 46]. Conversely, the cytokine antagonist infliximab, which blocks TNF, has been shown to have antidepressant properties, but only in depressed individuals with evidence of increased peripheral inflammation prior to treatment [47].

Elevated IL-6 in depression may be particularly relevant to cognitive symptoms. Increased peripheral levels of IL-6 cause increased production of IL-6 in the central nervous system (CNS) and affect neurogenesis in the hippocampus [reviewed in 48]. That the IL-6 is directly relevant to the changes seen is supported by several findings. First, these effects can be blocked by IL-6 receptor antagonists. Second, knockout mice with non-functional IL-6 genes have enhanced working memory compared with wild-type mice [49] and are refractory to peripheral inflammation-induced impairments of spatial memory [50]. In humans, raised levels of IL-6 are associated with diminished cognitive performance and reduced hippocampal gray matter [48, 51].

### Immunoregulation and psychiatric disorders in low-income countries

The vicious circle described in Fig. 2, considered against the background of the Old Friends mechanism, suggests that in low-income countries, there will be less release of inflammatory mediators in response to psychosocial stressors, and less psychiatric consequences of such stressors. It is important to note that the psychosocial stressors that are most depressogenic are remarkably constant across cultures [52]. They tend to involve social rejection, reduced status, loss of people or objects upon which one’s self-image depends and feeling trapped in one’s circumstances (entrapment). But effective immunoregulation should limit the release of inflammatory mediators in response to these universal human stressors. For example in a recent long term, longitudinal study performed in a low-/middle-income country, parental absence in childhood was a significant predictor of raised CRP in adulthood, as it would be in a high-income country (or in animal models [53]), but only in a subset of the cohort raised in hygienic environments early in life [54]. On the other hand, in this low-/middle-income country, adults who had a high level of microbial exposure in infancy were resistant to the long-term proinflammatory effects of this severe childhood stressor [54]. A similar pattern was seen for reports of perceived stress during the previous month in young adults. CRP correlated with recent perceived stress in the subjects with low microbial exposures in infancy, but not in those with high microbial exposures. These findings are consistent with the possibility that exposure to immunoregulation-inducing ‘Old Friends’ provided resistance to the inflammation-inducing effects of psychosocial stressors [54]. This leads to an obvious question. If psychosocial stressors cause depression at least partly by triggering the release of proinflammatory mediators (Fig. 2), then are inhabitants of low-/middle-income countries more resistant to stress-induced depression? If this is the case, then the prevalence of depression associated with raised biomarkers of inflammation should be increasing in developed countries and should be lower in developing countries than in developed ones. Interestingly, one study failed to find a correlation between depression and raised CRP in a low-/middle-income country [55], whereas this association is routinely found in high-income ones [56, 57], sometimes in association with other chronic inflammatory immunoregulatory disorders such as allergies, IBD and autoimmunity that are increasing in high-income countries, but rare in low-/middle-income ones [1, 2]. Ideally, we therefore need to distinguish between depression that is associated with raised biomarkers of inflammation, and depression that is not. Such data are not currently available, though several studies indicate that overall levels of depression are increasing in some high-income countries [58, 59]. Studies that compare high- and low-income communities are particularly difficult to conduct. The World Health Organization (WHO) and the World Bank try to gather such data [60]. These data show that a fall in intestinal helminths is linearly associated with a rise in World Bank income group (based on per capita Gross National Income), but that asthma, MS and depression show the reverse trend, becoming more rather than less prevalent as income increases [60]. This appears to be in good agreement with the Old Friends mechanism, but it is not clear that uniform methods and diagnostic criteria were used. More recently, the WHO World Mental Health Survey Initiative, using a common protocol and a common instrument (the WHO CIDI, version 3.0) found the highest prevalence estimates for depression in some of the wealthiest countries in the world (USA, France, New Zealand and The Netherlands). Similarly, lifetime prevalence estimates were found to be significantly higher in high- than in low- to middle-income countries overall [61], though there was wide variation within each income group. Clearly, further studies are required, as listed in Table 1, because a simultaneous fall in non-inflammation-associated depression could offset any putative increase in the inflammation-associated subset in some environments.

**Table 1.** Some unresolved questions, and tentative research approaches

Question or objective	Method
**Treatment**	
Can we use Old Friends and modulation of microbiota to treat chronic inflammatory disorders? (many trials in progress)	Immunoregulation-inducing ‘Old Friends’
	Appropriately selected probiotics
	Prebiotics
Can we use anti-inflammatory strategies to treat those psychiatric disorders that are accompanied by raised biomarkers of inflammation? (some trials in progress)	Anti-inflammatory drugs, COX2 inhibitors
	Cytokine inhibition (e.g. TNF (infliximab)) or block IL-6 or IL-1?
	Suppress activation of microglia (e.g. minocycline)
	Block MAPK or NFκB intracellular signaling pathways
**Prevention via environmental biodiversity**	
Can exposure to ‘Old Friends’ and environmental microbial biodiversity reduce the risk/prevent stress-related psychiatric disorders? Can such exposures reduce symptoms in currently afflicted persons and/or reduce the rate of symptomatic relapse in remitted individuals? Can we identify individuals with high risk (based on biomarkers) then treat prophylactically?	‘Domesticated’ helminths, or other immunoregulation-inducing Old Friends?
	Increased exposure to the natural environment
	Air-conditioning that introduces ‘Old Friends’ rather than *Legionella*
	Dish-washing or clothes-washing machines that introduce ‘Old Friends’
	Town planning that increases biodiversity in the environment
Is there a way to quantify exposure to Old Friends and microbial biodiversity?	Antibodies? Skin tests?
Is it beneficial to ensure neonatal exposure to maternal gut microbiota after cesarean delivery?	Epidemiology
**Green space**	
Is the green space effect due to microbial biodiversity acting via the immune system?	Interdisciplinary collaborations between epidemiologists, immunologists and microbiologists
Is resting CRP or IL-6 lower in people living near green spaces?	Interdisciplinary collaborations between epidemiologists and immunologists
Determine optimal content/species/soils/saprophytes, etc. for green spaces	Interdisciplinary collaborations between epidemiologists, immunologists, microbiologists, microbiome specialists and horticulturalists
Is exercise more beneficial when done in green spaces?	Compare health benefits of exercise in urban gyms with benefits of countryside jogging
**Epidemiology**	
Do SES gradients NOT correlate with health deficits in developing countries when confounders are eliminated?	New epidemiological studies
Is depression after influenza more likely if baseline IL-6 and CRP are high?	
Relative prevalence of depression with and without raised biomarkers of inflammation in low- and high-income countries, and rural versus urban environments	
Urban–rural differences in prevalence of ‘non-inflammatory’ disorders	
Urban–rural upbringing, proximity to green space, keeping a dog? … what are the effects on health gradients associated with SES?	Use existing databases (e.g. Whitehall studies) or new epidemiological studies
**Human experimental work**	
Is the pattern of pACC activation after a laboratory stressor seen in individuals who had an urban upbringing due to exposure to stress, or to reduced exposure to immunoregulatory Old Friends?	Measure cytokine responses to the stressor, and correlate with pACC activation. Compare with typhoid vaccine studies.
The role of the gut microbiota in the IL-6 response to the TSST or to typhoid vaccine	Test patients before and after they receive antibiotics that reduce gut microbiota

## EPIDEMIOLOGICAL PARALLELS BETWEEN INFLAMMATORY AND PSYCHIATRIC DISORDERS

If a dysregulated immune system resulting from diminished contact with immunoregulation-inducing ‘Old Friends’ is partly to blame for the increasing prevalence not only of chronic inflammatory disorders such as allergies, autoimmunity and IBD but also of those psychiatric disorders that can be triggered by inflammatory mediators, then it should be useful to examine urban–rural differences in disease prevalence, and the effect of migration from low-/middle-income to high-income urban environments. In each case, there will be loss of exposure to Old Friends. In the next sections, we consider these factors, and how they might interact with concomitant psychosocial stressors to determine vulnerability to psychiatric disorders, particularly affective disorders.

### Urban versus rural

A feature shared by most of the disorders discussed here is a higher prevalence in urban than in rural communities. This has been explored in some detail in relation to the allergic disorders. Contact with the farming environment, whether postnatal [6] or prenatal [62, 63], protects against allergic disorders, whereas the prevalence of these conditions increases with increasing urbanization [64]. The same is true for IBDs [65], and for autoimmune diseases such as MS [66, 67, discussed in 68]. These urban–rural differences are equally obvious in psychiatric disorders. For example, a meta-analysis of high-quality studies performed in high-income countries since 1985 found that the prevalence of depression in urban areas was 39% higher than in rural areas. Similarly, the prevalence of anxiety disorders was 21% higher in urban than in rural areas [69], though a small minority of studies fails to find this urban–rural difference [70]. Peen *et al.* [69] also noted an increased urban prevalence of psychiatric disorders in general (38% more in urban communities), and although outside the scope of this review, this is strikingly true for schizophrenia [71] and autism [72].

It has been suggested that vulnerable mentally ill people tend to gravitate toward socially deprived inner cities where deviant behavior might be more easily tolerated [73, 74], but data suggest that it is the urban upbringing rather than a selective migration into cities that lies behind the association of the urban environment with prevalence of psychiatric disturbance [75, 76]. Further light is cast on this point by considering immigration.

Does the increase in prevalence of these diseases in urban environments merely indicate that all diseases are more common in cities, or is there a specific urban-related increase in those with an inflammatory aetiology as the Old Friends mechanism would suggest? In the USA, a very high-income country, obesity and its associated problems are more prevalent in rural areas [77]. In New Zealand, asthma is more prevalent in urban communities, but cancer, migraine, stroke and COPD (smoking was similar in the two environments) are not [78]. These data are compatible with the view that there is a specific urban-related increase in disorders with an inflammatory aetiology that does not apply to non-inflammatory disorders, but clearly more studies that specifically target this question are needed (Table 1).

### Immigration, and age at immigration

All the diseases discussed here, whether chronic inflammatory [65, 79–81] or psychiatric [82–84], tend to be more common in immigrants than in the birth population from which these immigrants originated, at least when the migration is from a low-/middle-income to a high-income country.

#### Allergy

The role of migration in conferring risk for allergic disorders has been intensively examined. A study of children adopted into Sweden from low-/middle-income countries showed that the prevalence of asthma, hay fever and eczema was highest in those adopted when <2 years old [85]. Similarly, for Mexican immigrants to the USA, the prevalence of asthma was highest for those born in the USA, while in those not born in the USA, the prevalence of asthma decreased as the age at immigration increased [86]. This effect of age at the time of childhood immigration was also seen in immigrants to Israel from the former Soviet Union or Ethiopia who were assessed when 17 years old [87]. These observations suggest the importance of early environmental influences for allergy/asthma risk, a conclusion that is powerfully supported by evidence that prenatal exposure (i.e. of the pregnant mother) [62, 63] or early childhood exposure [6] to the farming environment protect the infant against some allergic manifestations.

#### Autoimmunity

Migration also has clear effects on the prevalence of MS [reviewed and referenced in 88, 89]. Iranians who migrate to Sweden have twice the prevalence of MS seen in their birth country [81]. Interestingly, if the second (or later) generation immigrants return to their low-/middle-income country of origin, they retain their increased susceptibility to MS, which remains higher than in the local population that was not born abroad [90]. Similarly, when people born in the UK (a high MS country) migrated to South Africa (a low MS country) they appear to retain the increased risk of their birth country rather than the lower risk of their new home [91]. The environmental factors that protect from or predispose to MS act during the first two decades of life [88, 89]. The same is true for T1D where the factor associated with elevated risk appears to be birth in the receiving developed country, again suggesting that the relevant environmental risk or protective factors act very early, or even in the prenatal period [80].

#### Inflammatory bowel disease

A definitive study of all first- and second-generation immigrants in Sweden between 1 January 1964 and 31 December 2007 showed that some first generation immigrants remain partially protected from both UC and CD, presumably by environmental factors encountered in their countries of origin, but the diseases increased in prevalence in second generation immigrants, relative to first generation immigrants [92]. Similarly, the prevalence of UC in South Asian immigrants to Leicester in the UK was higher in second than in first generation immigrants [93]. This again implicates perinatal or early life factors as potentially causative of this migration effect.

#### Psychiatric disorders

Depression is particularly interesting in this respect [94, 95]. Mexicans, Cubans and African/Caribbean peoples have a 2- to 3-fold increase in the prevalence of depression if immigration to the USA occurred when the individual was <13 years old, or was born in the USA, compared with the prevalence in those who migrated after the age of 13 years [94]. But this is not likely due to psychosocial stress related to skin color, because white Eastern European immigrants show the same effect. In sharp contrast, the effect is not seen in immigrants from Western Europe, or from Puerto Rico, which is closely associated with the USA (these last two populations already have a high prevalence of depression that is not increased by immigrating to, or being born in, the USA) [94]. These findings imply that influences important for depression occur perinatally, or in the early years of life.

Once again we mention in passing that the same is true for the psychotic disorders which are increased in immigrants, especially if they migrate when young, and increased further in second generation immigrants [96–98]. Autism is also strikingly increased in second generation Caribbean or African immigrants born in the UK [84].

### Diet and obesity

Migrant status and urbanization also affect the diet, which together with diminished exposure to biodiversity and elimination of helminths will alter the gut microbiome. Thus, the gut microbiome of Europeans was found to be strikingly different from that of people from a traditional rural village in Burkina Faso, and the differences were attributed to diet [33]. The nature of the microbiota profoundly affects immunoregulation [99]. Indeed, the microbiota constitute an important component of the immunoregulatory Old Friends with which we co-evolved, and many secrete molecules that drive expansion of the Treg populations as mentioned in Box 1. Diminished microbiome biodiversity in institutionalized elderly people correlates with diminished health and raised levels of peripheral inflammatory markers such as IL-6 [100], and poor or excessive diet can lead to obesity, which is also associated with distorted gut microbiota and increased peripheral inflammation [reviewed in 101]. It should however be remembered that some potently immunoregulatory Old Friends such as blood nematodes that are absent from high-income countries, never enter the gut [16], so the gut microbiota are only one, albeit important, component of the Old Friends.

## PERINATAL INTERACTIONS BETWEEN PSYCHOSOCIAL AND MICROBIAL FACTORS THAT MODULATE IMMUNOREGULATION

**Figure 3.**
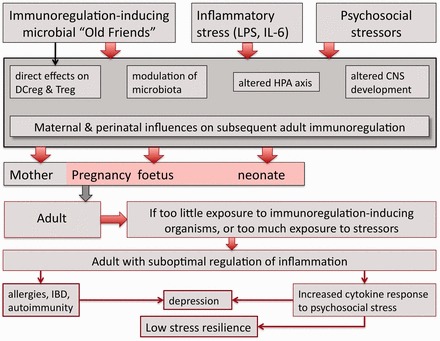
Perinatal influences on adult immunoregulation. Multiple factors in the perinatal period influence the developing brain, immune system, microbiota and HPA axis. Withdrawal of immunoregulation-inducing Old Friends and exposure to perinatal psychosocial stressors can result in immunoregulatory defects that are apparent in the adult. Such adults have increased risk of chronic inflammatory disorders, and increased inflammatory responses to psychosocial stressors, resulting in susceptibility to depression and probably to detrimental effects of low SES due to low stress resilience

The crucial importance of place of birth and age at migration highlighted in the previous section indicates that perinatal events and events in early childhood play a critical role in subsequent susceptibility to chronic inflammatory and psychiatric disorders (Fig. 3). Why might this be, and are there common pathways, perhaps converging on the control of inflammation, that explain these effects?

### Perinatal stress and long-term changes to immunoregulation and the brain

Immigrants from low- to high-income countries meet a changed microbial environment, with profoundly reduced biodiversity. They tend to be dewormed with anti-helminthics, while access to modern sanitation and reduced contact with contaminated soil lessen the risk of reinfection [102]. Thus, they lose contact with the Old Friends, but they also encounter a barrage of psychosocial stressors and these too can cause striking immunoregulatory problems in the perinatal period. Many studies in animals and humans have shown that stress during pregnancy activates inflammation [103, 104]. For example maternal stress during otherwise normal human pregnancies was associated with raised circulating levels of IL-6 and TNF, raised CRP and low levels of the anti-inflammatory cytokine IL-10 [105]. Similarly, overall stress levels during pregnancy correlated with increased release of IL-1β and IL-6 by maternal lymphocytes stimulated *in vitro* during the third trimester [106]. This is important because maternal immune activation during pregnancy [107, 108] or direct injection of IL-6 causes abnormal brain development in monkeys and rodents that can be opposed by IL-6 knockout, or by IL-6 neutralizing antibody [107]. Given these biological effects of inflammatory processes, it is perhaps not surprising that prenatal psychosocial stress (i.e. experienced by the pregnant mother) or early postnatal stress can cause long-term changes in neurogenesis [reviewed in 109], in cognition [110] and in HPA axis function in the offspring [111].

As a consequence of these long-term changes, adults previously exposed to perinatal stress themselves show exaggerated inflammatory responses to stress [112–115]. For example maltreated children develop higher levels of IL-6 in response to a standardized social stressor (TSST) when tested as adults in comparison to a non-maltreated control group [44, 112], and individuals maltreated as children tend to have higher levels of CRP 20 years later [114]. Low early life social class (SES) is similarly associated in adult life (aged 25–40 years) with increased production of IL-6 in cultures of peripheral blood leukocytes stimulated with ligands for toll-like receptor 3 (TLR3) or TLR5 [116].

These findings all imply that perinatal/early life stress leads to long-lasting problems with immunoregulation [112–114]. Interestingly, negative life events during the first years of life, whether they affect the child directly, or indirectly via traumatic experiences of the mother, also predispose to the autoimmune disease T1D later in life [117, 118, reviewed in 119]. It is likely that this reflects an influence of perinatal adverse life events on subsequent immunoregulation that is equally relevant to psychiatric and non-psychiatric chronic inflammatory disorders.

### Perinatal stress and long-term changes to the HPA axis

The HPA axis is a crucial immunoregulatory pathway (Fig. 3). Numerous animal models have demonstrated associations between prenatal stress and long-term alterations in HPA axis function [120, 121]. Observations consistent with this have been made in humans exposed to prenatal [111] or early childhood stress [122], or to a childhood background of low SES [116]. Moreover, adults with posttraumatic stress disorder (PTSD) symptoms who were abused as children show increased nuclear factor kappa-light-chain-enhancer of activated B cells (NFκB) and decreased glucocorticoid sensitivity and these two findings are highly correlated [123]. In monkeys, exposure to high levels of stress hormones *in utero* causes prolonged changes in the reactivity of the infant’s lymphocytes *in vitro* [124, 125]. These findings are consistent with the idea that HPA axis changes as a result of early abuse or neglect contribute to diminished regulation of inflammation.

### Perinatal stress and long-term changes to the intestinal microbiota

Changes to the microbiota impact the regulation of inflammation (Fig. 3). The nature of the microbiota is determined by the microbiota of the mother, and by the infant’s diet and environment [33] and by exposure to the Old Friends. But the microbiota are also modulated by stress [126]. For example, in rats and rhesus monkeys prenatal stress has effects on the microbiota that persist into adulthood [53, 127]. In humans, fluctuations in the microbiota early after surgery may lead to an increased risk of immunoregulatory failure, manifested as graft-versus-host disease [128], and changes in the microbiota of severely stressed critically ill humans are rapid and prolonged [129].

These effects of perinatal stress on the microbiota are important for two reasons. First, in animal models, the nature of the microbiota during the first weeks of life has effects on the development of the CNS and the HPA axis that persist into adulthood, and cannot be corrected by reconstitution of the microbiota of adult animals [130, 131]. Second, persistent alterations in the microbiota, whether due to lifestyle changes, urbanization, migration or perinatal stressors, also impact immunoregulation. The fundamental role of the microbiota (a major component of the ‘Old Friends’) in immunoregulation has been reviewed extensively elsewhere [19].

## THEORETICAL IMPLICATIONS

The previous sections suggest that factors such as migration and urbanization that alter exposure to ‘Old Friends’ (including microbiota) will interact with perinatal psychosocial stressors to modulate development of the brain, and in the longer term, to modulate the microbiota, the HPA axis and immunoregulation (Fig. 3). Crucially, these factors affect the immunoregulatory mechanisms that control susceptibility to chronic inflammatory disorders, and that appear to be relevant to the extent of release of psychoactive inflammatory mediators following psychosocial stressors (Fig. 2). (No doubt these immunoregulatory and psychosocial factors cause other changes too, e.g. changes to the sympathetic and parasympathetic systems, but these are beyond the scope of this review.)

In this final section, we provide examples to illustrate how considering this convergence of immunoregulatory and psychosocial mechanisms as set out in Figs 2 and 3 can change our perceptions of some psychiatric experiments and conditions, and change our interpretation of some recent experimental and epidemiological studies. We fully acknowledge the speculative nature of some of the suggestions we make.

### Depression in high- versus low-/middle-income countries

We considered earlier the evidence that there is a form of inflammation-associated depression, accompanied by raised CRP and IL-6, that is common in rich urban societies [56, 57], but probably rare in low-income countries [55]. In view of the forgoing discussion, we might view this as a ‘non-adaptive’ form of depression attributable to defective immunoregulation in communities deprived of immunoregulation-inducing ‘Old Friends’. This would be in sharp contrast to adaptive depression that possibly plays a role in driving appropriate changes in behavior. It would also change our perception of this defined subset of depressed individuals, and switch attention toward anti-inflammatory strategies.

### Stress response, immunoregulation and the ‘Old Friends’ mechanism

A recent functional magnetic resonance imaging (fMRI) study might also need to be re-interpreted in the light of the ‘Old Friends’ mechanism. This study compared the effects of an experimental social stressor on adults who had been brought up for all or part of their first 15 years of life in urban or rural environments. Urban versus rural upbringing correlated with significant differences in activation of the perigenual anterior cingulate cortex, a region involved in control of negative affect and the physiological stress response [132]. The authors attributed their findings to putatively different levels of social stressors in individuals with an urban versus rural upbringing. But would social stressors in children <15 years old differ significantly in the two environments in a wealthy European country (Germany)? The ‘Old Friends’ mechanism provides an alternative explanation because it predicts diminished regulation of proinflammatory mediators in those subjects who had an urban upbringing. Indeed, the protective effects of perinatal and early life exposure to the German farming environment against allergies and early onset IBD were discussed above [6, 133], and other recent studies that documented skin microbiota, atopic sensitization and *in vitro* release of an immunoregulatory cytokine indicate that immunoregulation might be enhanced by living within 2 or 3 km of agricultural land and forests, because of the impact on the immune system of the associated microbial biodiversity [30]. The authors of the fMRI study did not measure the stress-induced levels of circulating proinflammatory cytokines in the two populations. The ‘Old Friends’ view of the data would postulate higher levels of these cytokines in stressed subjects who had urban upbringings, so this issue is easy to resolve. Interestingly, it was shown previously that the subgenual anterior cingulate cortex, a component of the perigenual anterior cingulate cortex that was more activated by stress in those with an urban upbringing [132], is also activated in parallel with cognitive changes by an injection of typhoid vaccine, which provides a mild lipopolysaccharide-driven inflammatory stimulus and increased IL-6 [134]. This implies that the urban subjects in the fMRI study had a more inflammation-driven response to the stressor as predicted by the ‘Old Friends’ mechanism.

#### Stress resilience and gradients of SES

This argument can also be applied to the health gradients and background inflammation associated with gradients of SES. In the Whitehall study of UK civil servants, circulating levels of CRP and IL-6 were inversely correlated with employment grade, implying an inverse relationship between SES and background inflammation [135]. Moreover, raised CRP and IL-6 predicted subsequent risk of depression [39]. This and other studies show that the SES gradient is associated not only with biomarkers of inflammation but also with inflammation-mediated health deficits such as cardiovascular disease, that increase progressively at every rank below the top of the gradient, despite the fact that at these upper levels, diet and nutrition and healthcare access are not significantly different [136, 137]. A similar phenomenon has been seen in rhesus macaques that show a linear dominance hierarchy [138]. It is generally accepted that the inflammatory mediators are driven by psychosocial stress associated with low SES, but the arguments presented in this review suggest an additional level of control: the extent of the inflammatory response to stress will be partly controlled by immunoregulation (Fig. 2), and therefore by the ‘Old Friends’ mechanism [54]. In other words, the stress resilience of modern urban populations might be reduced because of poor background immunoregulation. Several additional points that lend weight to this view are discussed below.

First, the notion that any position below the top of a dominance or SES hierarchy is associated with long-term inflammation-mediated damage to health is difficult to reconcile with Darwinian medicine. Subordinate individuals later become dominant and play crucial roles as ‘leaders and breeders’ [reviewed in 137]. It might be maladaptive for such future breeding stock to receive permanent damage (for instance, to the cardiovascular system or IL-6-mediated damage to the hippocampus, cognition and memory) earlier in life. Most observations of stress and/or inflammation in subdominant animals have been made in populations that were captive or at least restricted by perimeter fencing. This will inevitably limit biodiversity and exposure to the faeces of other troops and species, and so partly deplete environmental ‘Old Friends’. Although in many troops of macaques or baboons, subordinate animals have high basal glucocorticoid levels, in other troops of the same species this effect is not seen [reviewed in 137]. We should ask ourselves whether the latter, more difficult to observe and record, is the adaptive situation, and the norm in thriving free-ranging communities. It is therefore conceivable that the steep slope of the SES-linked health deficit is a Western phenomenon, where social stressors are driving persistently elevated (and therefore damaging) levels of inflammation in the context of a dysregulated immune system (Figs 2 and 3). The work of McDade presented earlier already hints at the possibility that in a low-income country stress resilience is greatest in individuals who had high microbial exposures in childhood [54].

Second, although the confounders are so serious that we cannot yet compare populations from rich and developing countries, we can seek preliminary support for this notion in epidemiological studies that compare urban and rural communities in rich countries. This is reasonable because as explained above, we already know that a rural upbringing, or merely living close to agricultural land and forests, has demonstrable effects on immunoregulation and on chronic inflammatory diseases [6, 30, 133]. Interestingly, a recent very large study of the UK population confirmed a powerful link between SES (based on income group) and mortality (all cause or cardiovascular) [139]. But the slope of the health deficit gradient was strikingly less steep in subjects living close to green spaces. The same was observed in large studies in the Netherlands [140, 141]. All these studies imply that green spaces block the detrimental effects of low SES and that the effect is greatest at the lower end of the SES gradient, compatible with increased stress resilience [139–141]. The conventional explanations are a tendency to take more exercise when living close to green spaces (though this was not ascertained and may be untrue because people living near green spaces are often obliged to use their cars) or that contemplating trees is in some way psychologically beneficial, but we suggest that a better explanation is improved immunoregulation due to greater contact with ‘Old Friends’ and microbial biodiversity. It is curious that the large literatures on urban–rural differences [64–70], on the protective effects of the farming environment [6, 133], and the reduced atopy and increased biodiversity of the skin microbiota of people living within a few kilometers of agricultural land and forests [30] are not usually included in discussions of the benefits of exposure to green space, perhaps because they fall traditionally within different academic disciplines. However, these findings all point to a clear biological explanation for the beneficial effects of green space on health and wellbeing. If correct, this would have massive implications for the prevention of psychiatric and inflammatory problems in urban communities and would suggest several rather easy lines of research (Table 1) that might enhance our ability to re-introduce exposure to immunoregulation-inducing organisms, and enable us to supplement the ‘green space’ effect.

## FINAL REMARKS

In this review, we discuss the evolution of the immune system’s regulatory pathways and explore how the lifestyle of high-income countries may be leading to immunoregulatory deficits and uncontrolled inflammation that, in concert with psychosocial stressors, contribute to the rising tide of chronic inflammatory and psychiatric disease. The ‘bottom line’ is found in Fig. 2 where we illustrate diagrammatically the ways in which inflammation and psychosocial stressors, both equally valid and proven, interact and contribute to the changing patterns of disease in the modern world. The consequences are equally relevant to psychiatric disorders and to chronic inflammatory disorders.

This is not intended to be a comprehensive review. Had space permitted there are other psychiatric conditions that could have been included in the discussion because of evidence for inflammatory components: attention deficit hyperactivity disorder, and PTSD [142, 143], schizophrenia and autism [144, 145].

Similarly, we do not include a discussion of all the factors known to be relevant to associations between environmental conditions, immune function and physical and mental health. One additional factor of uncertain importance is delayed exposure to viruses caused by hygienic modern living conditions. Many viruses are harmless when met by neonates, perhaps because of the presence of maternal antibodies, but when encountered later such viruses may trigger inflammatory disorders such as allergies and autoimmunity [146–148]. Lack of vitamin D is also a feature of modern western lifestyles that has a major impact on immunoregulation and has been implicated in schizophrenia [149] as well as in several chronic inflammatory disorders [150–152], and exposure to modern pollutants such as dioxins might drive proinflammatory Th17 cells via the aryl hydrocarbon receptor [153].

In conclusion and with these limitations in mind, we suggest here that the pathways controlling brain development, stress responses and mood are so closely related to those controlling immunoregulation that they all need to be considered together. By loosening traditional interdisciplinary barriers in this review, we hope to have focused more attention on the relevance of psychosocial stressors in inflammatory disorders, and more attention on the potential for anti-inflammatory and immunomodulatory treatments for psychiatric ones. Finally, the conceptual framework that we provide suggests a number of areas where further research listed in Table 1 could rapidly cast additional light.
